# Demonstrating the data integrity of routinely collected healthcare systems data for clinical trials (DEDICaTe): A proof-of-concept study

**DOI:** 10.1177/14604582241276969

**Published:** 2024-07-01

**Authors:** Macey L Murray, Laura Sato, Jaspal Panesar, Sharon B Love, Rebecca Lee, James R Carpenter, Marion Mafham, Mahesh KB Parmar, Heather Pinches, Matthew R Sydes

**Affiliations:** MRC Clinical Trials Unit at UCL (MRC CTU), Institute of Clinical Trials and Methodology, UCL, London, UK; Health Data Research UK (HDR UK), London, UK; NHS DigiTrials and Research Products Services, Data & Analytics, NHS England (NHSE), Leeds, UK; Corporate Metadata Team, Transformation Directorate, NHS England, Leeds, UK; MRC Clinical Trials Unit at UCL, Institute of Clinical Trials and Methodology, UCL, London, UK; Health Data Research UK, London, UK; Corporate Metadata Team, Transformation Directorate, NHS England, Leeds, UK; MRC Clinical Trials Unit at UCL, Institute of Clinical Trials and Methodology, UCL, London, UK; Health Data Research UK, London, UK; Department of Medical Statistics, London School of Hygiene and Tropical Medicine, London, UK; Nuffield Department of Population Health, University of Oxford, Oxford, UK; Health Data Research UK, London, UK; MRC Clinical Trials Unit at UCL, Institute of Clinical Trials and Methodology, UCL, London, UK; Health Data Research UK, London, UK; NHS DigiTrials and Research Products Services, Data & Analytics, NHS England, Leeds, UK; MRC Clinical Trials Unit at UCL, Institute of Clinical Trials and Methodology, UCL, London, UK; Health Data Research UK, London, UK; British Heart Foundation Data Science Centre, HDR UK, London, UK; Data for Research and Development Programme, Transformation Directorate, NHS England, London, UK

**Keywords:** clinical trials, data integrity, data quality, data provenance, healthcare systems data, metadata, routinely collected health data

## Abstract

**Introduction/aims:**

Healthcare systems data (also known as real-world or routinely collected health data) could transform the conduct of clinical trials. Demonstrating integrity and provenance of these data is critical for clinical trials, to enable their use where appropriate and avoid duplication using scarce trial resources. Building on previous work, this proof-of-concept study used a data intelligence tool, the “Central Metastore,” to provide metadata and lineage information of nationally held data.

**Methods:**

The feasibility of NHS England’s Central Metastore to capture detailed records of the origins, processes, and methods that produce four datasets was assessed. These were England’s Hospital Episode Statistics (Admitted Patient Care, Outpatients, Critical Care) and the Civil Registration of Deaths (England and Wales). The process comprised: information gathering; information ingestion using the tool; and auto-generation of lineage diagrams/content to show data integrity. A guidance document to standardise this process was developed.

**Results/Discussion:**

The tool can ingest, store and display data provenance in sufficient detail to support trust and transparency in using these datasets for trials. The slowest step was information gathering from multiple sources, so consistency in record-keeping is essential.

## Background

Healthcare systems data (HSD), otherwise referred to as real-world data or routinely collected healthcare data, have increasingly-recognised value for medicines research and regulation.^1–3^ They have mostly been used in observational research, such as disease epidemiology and post-authorisation safety studies, but several recent high-profile clinical trials have shown how HSD can support, or even replace, trial-specific data collection.^4,5^ In recognition of this important shift in trial design and management, medicines regulators in the UK, USA and Europe have separately developed guidance for HSD use in clinical studies for regulatory decision-making, as well as encouraging further assessment of the quality of HSD.^2,3,6^

Clinical trial sponsors must demonstrate that all data used in a clinical trial have integrity, are reliable, and complete.^7,8^ This includes healthcare systems data which may be used to support participant recruitment and inform trial outcomes. The UK medicines regulator, the Medicines and Healthcare products Regulatory Agency (MHRA) state in their guidance on real-world data that the quality of the data “*should be understood including its accuracy, validity, variability, reliability and provenance*,” and in particular that “*processes are established to ensure the integrity of the data from acquisition through to archiving*.”^2^ Hence, data providers must supply detailed information on the provenance and integrity of their datasets to trialists, regulators, as well as other researchers. Provenance is the detailed records of the origins of the data, the processes and methods used to produce it. Integrity is the extent to which the data are complete, consistent, accurate, and reliable throughout the data lifecycle. The MHRA states in its GXP data integrity guidance that “*the controls required for integrity do not necessarily guarantee the quality of the data generated*.”^9^ Hence data integrity should not be confused with data quality and matters of how suitable is the dataset for its intended use, sometimes referred to as “fitness for purpose,” and is the responsibility of the data user to justify.

A manual process to ascertain and document provenance and integrity was previously established.^10,11^ It showed that two datasets frequently requested by trialists, NHS Digital’s (now NHS England [NHSE]) Admitted Patient Care of Hospital Episodes Statistics (HES APC) and the Civil Registration of Deaths (CRD), are both high-quality, transcribed copies of the original source data. HES APC covers all in-patient admissions in NHS hospital trusts in England and CRD contains all registrations of death certificates issued in England and Wales. However, that assessment referenced many sources of information so would become outdated with changes to the data lifecycle or the reference information.

Publication of the FAIR principles (Findable, Accessible, Interoperable and Reusable) and the increasing availability of metadata catalogues is facilitating health research and regulatory decision-making by standardising metadata information and aiding discovery of appropriate data.^12^ Examples include the Health Data Research (HDR) Innovation Gateway, the Minerva project pilot, and the proposed EU Real World Metadata Catalogue.^13–15^ Furthermore, metadata tools are valuable, not just for research, but some can also support data governance within organisations. Examples of such tools are Collibra and Mauro Data Mapper (open source, developed by the University of Oxford).^16,17^

NHSE’s data intelligence tool, the Central Metastore, is being used to capture metadata and data lineage, and is intended as their single source of truth about the data they hold. This repository can facilitate documentation of the data lifecycle that can be updated in real-time (or close to real-time) when there are modifications in data management, for example, the addition of a new processing rule, or changes to business rules for extraction from healthcare providers. It can do this by automatically “ingesting” metadata directly from a data platform source; this function will enable NHSE to document and centrally manage metadata of >200 data assets,^18^ providing an umbrella view of all its data. The system is open and capable of reflecting any data model standards, and represents data models from HL7 FHIR resources, OMOP (Observational Medical Outcomes Partnership) and the NHS Data Model and Dictionary^19^ for reference and correlation to national data set data models. It also has an Open API available for communicating metadata with other systems via its back end. The DEDICaTe study (Demonstrating the Data Integrity of routinely collected healthcare systems data for Clinical Trials) set out to test as a proof-of-concept whether the Central Metastore could semi-automate capture of provenance and integrity information of four national datasets, and to automate the generation of lineage diagrams in a coherent format for data users. The provision of diagrams and explorable metadata sought to improve transparency and thereby support trust (and show limitations) in using these data for clinical trials and health research.

## Methods

Data provenance, a type of metadata, can be conceptualised in a data model to describe the production of data – who and what are involved, so that their quality and reliability can be evaluated.^20^ This proof-of-concept study assesses the feasibility of using NHSE’s Central Metastore to semi-automatically ingest metadata and provenance components to produce data models and lineages. We describe our approach and experience.

We selected four datasets to test our proof-of-concept: two were the datasets held by NHSE included in the original position paper on data integrity,^10^ HES APC and CRD. The other two were the Outpatients (OP) and Critical Care (CC) datasets of HES as they have been of particular interest to trialists and health economists.^21,22^ The datasets that underpin HES were originally designed for reimbursement and commissioning purposes but are valued for research.

NHSE’s Central Metastore can store and inter-relate all types of information about data, not only their characteristics and meaning, but also the business terms and processing rules applied to the data, where data are stored and accessed, and who owns and controls them. Once this information is stored in the platform, the system is able to auto-generate diagrams to illustrate how data relate to business services and processes (‘business lineage’). It can also show how data move between different representations via pipelines (‘data lineage’) and how data move between physical locations (‘technical lineage’). This study focused on providing clinical trialist data users with combined business, data and technical lineages of the four prioritised datasets.

Three steps were necessary in the preparation and production of provenance and lineage information in the Central Metastore. First, gathering this information, which included the logical data dictionary, and the rules at each of the three stages in the data lifecycle (submission: collection from healthcare providers; production: processing and curation within NHSE; and release: linkage and extraction for the end user). Second, using the Central Metastore to ingest information to form complete data models, including the business rules (i.e., extraction specification), processing, derivations, data quality and validation rules. Third, using the Central Metastore to automatically generate lineage diagrams and content (such as reports or catalogue views) to provide information on provenance for data users and regulators.

A key output was the development of an openly available operating manual as guidance for NHSE staff on populating the Central Metastore. This was imperative so that metadata of other NHSE datasets could be ingested in future, and to allow sharing of the process with other data providers who want to do similar work.

## Results

The gathering of lineage information and data ingestion of the four NHSE datasets were initiated in June 2022 and completed by December 2022. The time required for information gathering was longer than anticipated because NHSE had not yet implemented automation for the collection of metadata from its (multiple) data platforms. For the Hospital Episode Statistics datasets, full lineage information was difficult to find as these datasets were initiated in 1989, and the pipelines were developed piecemeal over time and involved multiple data processing platforms within NHSE. The provenance and lineage information for these datasets had not been held in a single place prior to this project. Therefore, metadata had to be obtained from documentation held by specific technical teams within NHSE, and then prepared in a structured format for ingestion.

**Figure 1:**
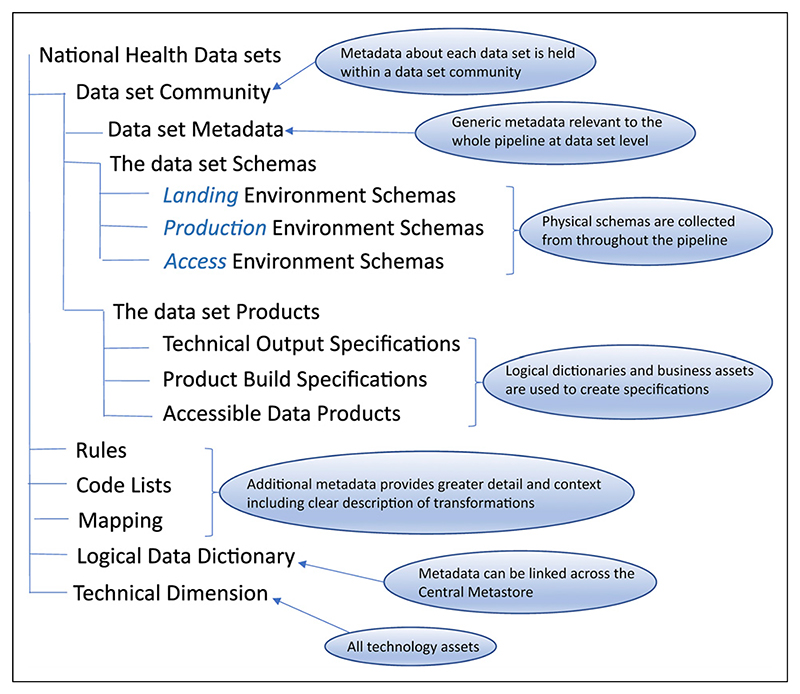
The elements of the metadata model captured in NHS England’s Central Metastore, reproduced from the study’s operating manual/guidance.^23,24^

The newly-generated operating manual (guidance) is available from NHS England’s DEDICaTe website.^23,24^ It describes generic and tool-specific data lineage development including which information is required and the method for lineage development, contact with key teams in the submission-to-dissemination pipeline, acquisition of key specifications, schema information and key parties, identification of data transformations, and ingestion of relevant schemas and specifications, such as associated rules, code lists, and mapping.^23,24^ Figure 1 shows the elements of the metadata model that are captured in the Central Metastore. The tool-specific section of the guidance gives details of how to create the technology asset domain, data models, rulebooks, and mapping specifications of the datasets, and refers to the structured templates used to ingest the information.^24^

**Figure 2:**
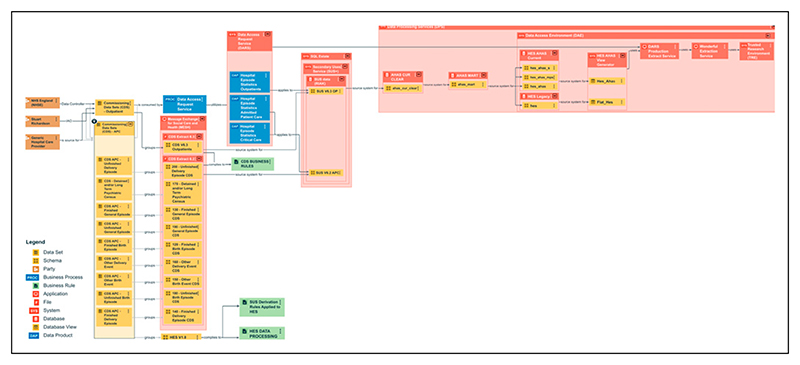
Summary business lineage view of the Admitted Patient Care (APC), Outpatients (OP), and Critical Care (CC) datasets of Hospital Episode Statistics (HES).^23^ The data journey from left (submission from hospitals using business rules via an XML schema) to right (production stage where derivations and processing rules are applied) to form the final releasable HES schema containing APC, OP and CC tables in the data access environment (far right).

**Figure 3:**
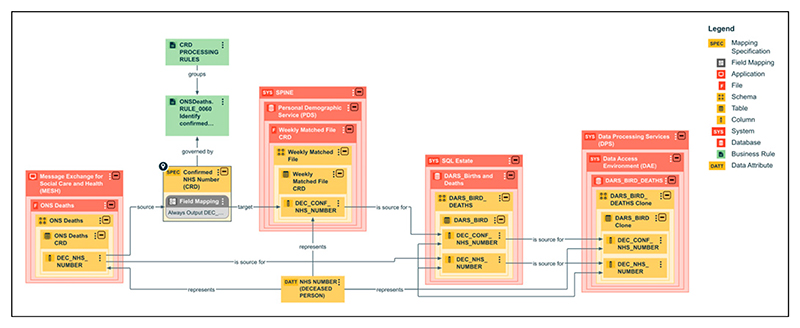
Field level lineage of the Civil Registration of Deaths (CRD) with an example of derivation to confirm NHS number.^23^ The data journey moves from left (submission via Message Exchange for Social Care and Health) to right (production stage, then to releasable in the data access environment).

**Figure 4:**
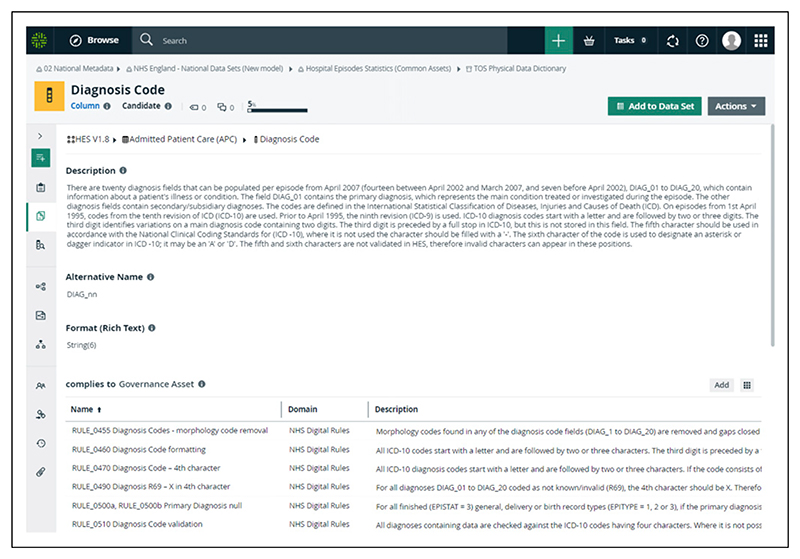
Example of data item details captured in the Central Metastore at NHS England (diagnosis code, Hospital Episode Statistics Admitted Patient Care).

The process now presents the provenance of each of the four datasets (CRD, HES APC, OP and CC) characterised in the Central Metastore; each dataset comprised 158, 722, 371 and 123 data elements respectively. The complete lineage (business, data, and technical) of the four datasets were captured and detailed diagrams generated (Figures 2 and 3). The diagrams in the Central Metastore are interactive, so further information about specific assets or data fields can be accessed by selecting the item. Catalogue views of the metadata (the logical data dictionary view) can be used, which allow users to search the names of data items and assets and to see which datasets include the data item along with the names of the physical columns mapped to that item. Detailed information about each data item or asset is available, such as descriptions of the rules that it complies to; these rules are numbered to allow cross-referencing to the published HES Technical Output Specification (also previously known as the HES Autocleans Dictionary) (Figure 4).^25^ Referencing external documentation or key sources of information in the Central Metastore (under the term “associated media”) allows full evidence of provenance and integrity to be captured, so pertinent publications from ONS on the management of CRD data were referenced.^26,27^ Data quality metrics, such as completeness and validity of data items, which are published monthly by the Data Quality team (as the Data Quality Maturity Index) are not currently available in the Central Metastore, but reference to these published reports is possible.^28^

The Central Metastore is able to generate summary and detailed diagrams to show data flows, such as the lineage of specific data fields, copies of which can be provided for storage in the Trial Master File of each trial using the dataset. Figures 2 and 3 are examples of the type of information provided in the diagrams: Figure 2 is a summary view of the business lineage of the HES datasets; Figure 3 illustrates the derivation required to confirm NHS number in the processing of CRD. The business lineage view of HES shows how business, physical and technology assets relate so as to describe the data journey such as the parties involved (original source of the data, Information Asset Owner, Data Controller). In particular, HES APC, OP and CC have common lineage during the submission stage from hospitals as they are extracted using the same business rules (XML schema) to produce the Commissioning Datasets (CDS) extracts from hospital providers submitted to NHSE through the secure document transfer protocol, Message Exchange for Social Care and Health (Figure 2). The diagram shows that there are two CDS versions currently in use (versions 6.2 and 6.3). The derivation and processing rules applied to form the final HES schema containing APC, OP, and CC data tables can be seen in green in the figure as “SUS Derivation Rules Applied to HES” and “HES Data Processing Rules.” The arrows denote relationships between schemas (“source system for”), and show the applications used to view or extract data (“uses”) in the Data Access Environment.

CRD has fewer processing rules within NHSE because the majority of the derivations and validations are conducted by the Office for National Statistics in generating the source data and providing them to NHSE.^26,27^ Figure 3 shows that NHS number from the ONS data file (DEC_NHS_NUMBER) is validated via the Personal Demographics Service (PDS; the national master database of all NHS patients in England, Wales and the Isle of Man). A second NHS number (DEC_CONF_NHS_-NUMBER) is included with the data and will match the original number if it is validated by PDS. Similar to HES data, the final CRD data product is held in the Data Processing Services platform within the Data Access Environment, accessed using the same applications as HES.

A new website (https://dedicate.healthandcaremetadata.uk) has been developed to provide open access to outputs from this project, and currently comprises summary and field-level lineage diagrams of the four datasets with examples of derivation, code list, validation and data removal (e.g. for invalid discharge date).^23^ It is also possible to request access to the logical dictionary view of the Central Metastore via the website to explore the lineages of the four datasets in more detail. Two recorded videos (in the section “Video demo overview”) lead viewers through the lineages of the Civil Registration of Deaths and Hospital Episode Statistics.^23^

## Discussion

### Main findings

In this first proof-of-concept, and only exact type of study in the UK so far, we have shown that NHS England’s Central Metastore is a feasible tool for recording data provenance and integrity information within a centralised repository, producing complete provenance and lineage models of the four key national datasets. Demonstrating the integrity of a dataset involves qualitative review of its complete lineage, processing and methods of production to confirm that data are handled reliably, and remain accurate, complete, and consistent through their lifecycle. Detailed information captured within the Central Metastore allows integrity to be qualitatively shown especially as it allows reference to data quality reports produced internally and published reports such as ONS documentation. The data user can easily review the data validations and derivations used to form the final dataset; an example is the confirmation of NHS number in the Civil Registration of Deaths (Figure 3). The accessible Central Metastore aids transparency about the management and production of the four datasets by NHSE, building public trust in research that uses these reliable data, especially clinical trials. Trial participants, the public, funders, and healthcare providers can be assured of NHSE’s handling of health data, with robust controls and processes. Responsibility for determining whether HSD are suitable for a clinical trial always remains with each trial’s sponsor, with the rationale recorded in the Trial Master File, using a document like the routine dataset justification template.^29^ When using a NHSE-held dataset, a sponsor will be able to turn to this rich resource for reference in contributing to their own assessment over the relevant lifespan of the trial. The summary or field level data flow diagrams are available to data users as required, and examples from the four datasets provided on the DEDICaTe webpage.^23^ This first necessary step of making NHSE data lineage information available through the Central Metastore to data users, specifically clinical trialists and regulators, should encourage other data providers to do similarly in future. An important next step to support this work is the user design of customised views into NHSE’s Central Metastore that provide sufficient technical detail to demonstrate the data provenance and integrity of individual HSDs.

### Limitations

The Central Metastore is a suitable central repository and is capable of automating the creation of provenance and lineage diagrams from the information it holds. However, automating the capture and ingestion of metadata from data platforms and other relevant systems with information about the dataset requires system-to-system interfaces that have not yet been implemented at NHSE at the time of the study. Therefore, one limitation of this approach is the current need for manual preparation and ingestion of metadata, which could result in incomplete or incorrect lineage information. The operating manual/guidance describes the detailed manual steps which will mitigate possible errors. Once enabled, the Central Metastore can maintain complete up-to-date lineage models of existing NHSE datasets, especially if a data pipeline or a processing rule changes, as well as ingest information about new datasets. Data holders/custodians should consider the ‘end-to-end’ process requirements for automation in their tools and systems infrastructure planning (including necessary joins between systems) in order to reduce the manual effort required to communicate full data lineage information from multiple systems into a central metadata repository.

The delays experienced during this project were caused by inconsistent and missing documentation about pipeline processing and data storage forms and locations, particularly for the HES datasets, which have been held at NHSE for much longer than the CRD. It would have aided the project immensely if this information was already held in a single source or if standards for documentation of this information were in place across all pipelines and platforms teams. This is another limitation to the process, and impacted the efficiency of information gathering, so consistency in record-keeping and capture of data provenance/lineage is imperative.

Another limitation is the function to produce detailed lineage diagrams that interactively drill down to the field level is unique to Collibra, the commercial software product currently used in delivering NHS England’s Central Metastore service. While opportunities may exist for sharing this tool and the metadata platform across UK data providers in future, we strongly encourage developers of open-source metadata tools to embrace this functionality, as this will help a wider selection of data providers to make available this type of information about their data. For example, a recent study within a German medical data integration centre developed a Python library to capture data provenance, and produced visualisations using the Mermaid plotting framework.^30,31^

### Further work

Further work is ongoing more widely to facilitate the documentation of data provenance and integrity of other HSD, including those at NHS England (such as the Systemic Anti-Cancer Therapy dataset), and to make the information available to all data users, including trial sponsors and regulators. It is important to collate this information consistently, using clearly defined standards or metrics so a sponsor can assess whether or not to use a particular HSD for its trial (along with its assessment of access costs, timeliness, utility, ability to onward-share and retain). Currently, the European Medicines Agency’s guide on metadata catalogues recommends the inclusion of descriptors of data flow and management, such as governance, data quality and validation check results, and the systems used to gather and store data.^15^ The expert insights of data holders, trial sponsors, and regulators are needed to help develop standard criteria around recording data provenance and integrity, to ensure the criteria satisfy regulatory requirements and are pragmatic for data providers to follow.

## Conclusion

This unique study shows how a centralised, semi-automated repository can produce complete provenance and lineage models of four national datasets frequently used to obtain outcome data for clinical trials. The quality bar for using any data in trials, including HSD, is set higher than for other forms of research, so straightforward access to maintained information on data integrity and provenance is necessary to build trust and unlock the transformative use of these datasets. This first key step of making NHSE data lineage information available through the Central Metastore to data users, including clinical trialists and regulators, should be followed by other data providers in the UK and more widely.
